# Two-Step Isolation, Purification, and Characterization of Lectin from Zihua Snap Bean (*Phaseolus vulgaris*) Seeds

**DOI:** 10.3390/polym11050785

**Published:** 2019-05-02

**Authors:** Bin Jiang, Xiaojing Wang, Linlin Wang, Xiaomeng Lv, Dongmei Li, Chunhong Liu, Zhibiao Feng

**Affiliations:** Department of Applied Chemistry, Northeast Agricultural University, NO.600 Changjiang Road Xiangfang District, Harbin 150030, China; jiangbin@neau.edu.cn (B.J.); neauwxj@163.com (X.W.); neauwll@163.com (L.W.); 18846173287@163.com (X.L.); lidongmei@neau.edu.cn (D.L.); liuchunhong@neau.edu.cn (C.L.)

**Keywords:** Zihua snap bean, lectin, stability, preliminary property, bioactivity

## Abstract

A two-step method based on an aqueous two-phase system and Sephadex G-75 was used to separate and purify lectin from the seeds of the Zihua snap bean. The preliminary properties and bioactivity of the Zihua snap bean lectin were characterized by different instrumental methods, such as sodium dodecyl sulfate polyacrylamide gel electrophoresis (SDS–PAGE), liquid chromatography-nano electrospray ionization mass spectrometry (Nano LC-ESI-MS/MS), and Fourier transform infrared spectroscopy (FTIR). The hemagglutinating activity of the Zihua snap bean lectin could not be inhibited by glucose, *N*-acetyl-d-glucosamine, d-galactose, *N*-acetyl-d-galactosamine, fructose, sucrose, d-maltose, d-trehalose, and lactose. It was found that the hemagglutinating activity of the lectin showed strong dependence on Mn^2+^ and Ca^2+^. The thermal and pH stability of the Zihua snap bean lectin was studied by FTIR and fluorescence spectroscopy. Relatively good stability was observed when the temperature was not higher than 70 °C, as well as in the pH range of 2.0 to 10.0. Digestive stability in vitro was investigated. The untreated lectin was relatively stable to pepsin and trypsin activity, but heat treatment could significantly reduce the digestive stability in vitro. Moreover, the lectin showed an inhibitory effect on the tested bacteria (*Staphylococcus aureus* (*S. aureus*), *Escherichia coli* (*E. coli*), *Bacillus subtilis* (*B. subtilis*)), and it also showed a certain inhibitory effect on the growth of *Phytophthora infestans* (*P. infestans*) at higher concentrations.

## 1. Introduction

Lectins are glycoproteins that are characterized by their capability to attach carbohydrates such as mannose, galactose, lactose, *N*-acetyl glucosamine, *N*-acetyl galactosamie, fucose, and rhamnose with significant specificity [1]. They bind specifically and reversibly to different types of glycoproteins or carbohydrates [2,3]. Due to these advantages, some sensitive dielectric sensors based on Concanavalin A lectin were used for the specific identification of glycoproteins and carbohydrates [4,5]. 

Exploratory studies on their health benefits have been initiated, because lectins have important physiological roles, including insecticidal action [3,6], antibacterial activity [7], and antifungal effects [8], antihuman immunodeficiency virus [9], antitumor activity [10,11,12], and analgesic activity [13]. Plants are the main source of lectins that are found in different parts of plants such as seeds, leaves, bark, roots, tubers, and fruits [14]. It has been known for a long time that lectins occur in legumes, where they can be a major food source for both humans and animals [15]. However, lectin has an antinutritional factor [16]. Its antinutritional properties are most likely caused by their impairment of the integrity of the intestinal epithelium, and thus also the absorption and utilization of the nutrients that are present in the legumes [17]. So, lectins containing foods are frequently consumed cooked or otherwise processed to reduce the level of the antinutritional factors and improve the utilization efficiency of legumes [18]. In addition, the lectins that are thought to survive gastric digestion have been a minor allergen, such as soybean seeds [19,20]. Therefore, its relevance to food safety requires intensive research to determine the digestibility of lectins from legumes, especially regarding the consumption of high levels of unprocessed or undercooked beans [21]. Thus, more and more legume lectins are being studied intensively. Lectin with a molecular mass of approximately 60 kDa and two different subunits was isolated from the ground bean (*Vigna sesquipedalis* cv. ground bean) [22]. Lectin with a molecular mass of 67 kDa, and two identical subunits, was purified from *Phaseolus vulgaris (P. vulgaris)* cv. dark red kidney bean [23]. He et al. [24] extracted a lectin with the molecular weight of 49.24 kDa from small black kidney bean (*P. vulgaris*) using a reversed micellar system. 

The Zihua snap bean is a high quality bean that is distributed in northeast China. The protein content in Zihua snap bean seeds exceeds 20% [25], of which 2.4–5% is lectin [15]. In our previous studies, lectin was separated from the Zihua snap bean (*P. vulgaris*) seeds by an aqueous two-phase system (ATPS), which was effectively applied for the extraction and purification of proteins and other biomolecules [26,27,28].

In this study, seeds of the Zihua snap bean, which is an endemic species in northeast China, were chosen to separate and purify lectin by a two-step method based on an aqueous two-phase system and Sephadex G-75. On this basis, the partial properties of lectin were investigated. In addition, the in vitro digestion characteristics and antimicrobial activity of the lectin were also discussed. The purpose of the present work was to establish a two-step method for purifying lectin from the Zihua snap bean in local special crops and explore new sources of lectin. Furthermore, system information about the reduction of antinutritional factors of lectin by heat treatment and the effect of lectin preheating on digestion were provided. These laid the foundation for the deep processing of the Zihua snap bean to improve the economic value of the Zihua snap bean.

## 2. Materials and Methods 

### 2.1. Instruments

An ultraviolet-visible spectrophotometer was from Beijing Purkinje General Instrument Co., Ltd. (Beijing China). An A150011 vortex mixer (Nanjing Jiajun Biological Co., Ltd., Nanjing, China) and a SC-3610 low speed centrifuge (Anhui Zhongke Zhongjia Scientific Instrument Co., Ltd., Hefei, China) were applied to treat the sample. The Biorad Mini-PROTEAN Tetra Cells 4-Gel 165-8004 was from Bio-Rad Co., Ltd. (Guangzhou China). The pH of solution was measured by an FE201EL20 pH meter (Yidian Scientific Instrument Co., Ltd., Shanghai, China), and the weight of the sample was determined by an AL-04 electronic analytical balance (Mettler Toledo Instruments Co., Ltd., Shanghai, China).

### 2.2. Reagents

Zihua snap bean (*P. vulgaris*) seeds were from Harbin Xiangfang District Gongbin Seed Company (Harbin, China). A 2% rabbit red blood cell suspension was obtained from Beijing Baiaolaibo Technology Co., Ltd. (Beijing, China). All the chemicals were obtained from Aladdin (Shanghai, China) and were of analytical grade, and all the solutions were prepared using ultrapure water obtained from Northeast Agricultural University.

### 2.3. Extraction and Purification of Zihua Snap Bean Lectin

Dried Zihua snap-bean seeds were ground to a fine powder passed through a 50-mesh sieve. Then, the powder was mixed with phosphate-buffered saline (PBS, 10 mM, pH 7.0) by agitation overnight at 4 °C. Afterwards, the extract was filtered and centrifuged to get a supernatant as the crude extract. 

The crude extract was purified using the aqueous two-phase system (ATPS) method as previously described [25]. ATPS was formed by mixing 0.75 g of ammonium sulfate, 0.9 g of polyethylene glycol 600 (PEG 600), and 0.4 g of NaCl, adding 1 mL of crude extract solutions. The total weight of the system was 5 g with the pH set on 7.5. After separation by ATPS, lectin and proteins in the top phase were collected and dialyzed against deionized water for 24 h by a dialysis bag with 7000-Da molecular weight cutoffs to remove salt and PEG 600. 

The lectin was further purified by gel chromatography. A 10 mg/mL lectin solution was prepared by concentrating the dialyzed lectin solution and then filtering it through a 0.45-μm membrane. Then, 5 mL of the filtered liquid was applied to a Sephadex G-75 column pre-equilibrated with the 0.02-M phosphate buffer (pH 7.2). The column was washed with eluent at a flow rate of 0.5 mL/min. The peak fraction prepared for the determination of hemagglutinating activity was collected and pooled, and then dialyzed and lyophilized to obtain lectin lyophilized powder for use.

### 2.4. Determination of the Hemagglutinating Activity

The determination of the hemagglutinating activity (HA) was performed in microtiter plates according to Jiang [25]. The lectin lyophilized powder was formulated into lectin solution (0.5 mg/mL). Then, 50 μL of lectin solution was twofold serially diluted with stroke-physiological saline, and 50 μL of 2% rabbit red blood cell suspension were added. The results were read after 45 min at room temperature, when the negative control was fully sedimented. 

The sugar specificity of lectin was analyzed in a manner analogous to the hemagglutination test. The sugar base to be tested was formulated into a certain concentration of aqueous solutions. Equal volumes (25 μL) of the sugar solution and the lectin solution were mixed, and stored at room temperature for 1 h. Then, the mixed solutions were diluted to detect hemagglutinating activity. Water was set as a blank control to observe the inhibition of the lectin hemagglutinating activity by different glycosyl groups. Each experiment was repeated three times.

### 2.5. The Properties Experiment of the Zihua Snap Bean Lectin

#### 2.5.1. Metal Ions Dependence

The lyophilized powder of the Zihua snap bean lectin was dissolved in 10 mmol/L phosphate buffer (pH 7.2) to prepare a lectin solution of 0.5 mg/mL, and the hemagglutinating activity of lectin solution was determined. Lectin solution was dialyzed in a phosphate buffer solution (10 mmol/L, pH 7.2) containing 50 mmol/L ethylenediaminetetraacetic acid (EDTA) until no hemagglutinating activity was detected. Then, it was dialyzed against phosphate buffer solution to remove EDTA, and the obtained sample solution was subjected to a hemagglutinating activity analysis to confirm that the hemagglutinating activity was lost. Lectin solution without hemagglutinating activity was mixed with different metal salt solutions (100 mmol/L), and then diluted to detect hemagglutinating activity to study the effect of metal salt ions on the recovery of hemagglutinating activity. Each experiment was repeated three times.

#### 2.5.2. Thermal Stability of Zihua Snap Bean Lectin

A thermal stability experiment followed He’s method with minor modification [29]. The method of lectin solution configuration was the same as in Section 2.5.1. The lectin solution in 10-mL centrifuge tubes was heated to the desired temperature using a constant temperature water bath. All the tubes were completely submerged in the water bath throughout the investigation and ensured to prevent the evaporation of water or loss of solution. A series of thermal treatments were carried out at 50 °C, 60 °C, 70 °C, 80 °C, and 90 °C for 0 min, 5 min, 10 min, 15 min, 20 min, 25 min, and 30 min. At the end of the thermal treatment, it was immediately cooled in cold water and stored at 4 °C until further analysis.

#### 2.5.3. pH Stability of Zihua Snap Bean Lectin

The lectin lyophilized powder was dissolved in buffers of desired pH to formulate a solution of 0.5 mg/mL. After standing at 4 °C for 12 h, the acid–base stability of the lectin was studied by a hemagglutinating experiment and fluorescence spectroscopy, respectively. Glycine–HCl buffer solution at pH 2.0, citrate buffer solution at pH 3.0 to 5.0, phosphate buffer at pH 6.0 to 8.0, and glycine–sodium hydroxide buffer solution at pH 9.0 to 11.0 were used to maintain the pH.

#### 2.5.4. In Vitro Digestion Assay of Zihua Snap Bean Lectin

Simulated gastric fluid (SGF) was prepared according to He [21], which consisted of 2.0 g/L of NaCl and 3.2 g/L of pepsin. The pH value of the SGF solution was adjusted to 1.2 with hydrochloric acid. Lectin solution (5 mg/mL) was mixed with the SGF solution (pH 1.2) in a water bath at 37 °C to start the digestion reaction. The pepsin reaction was terminated by adding Na_2_CO_3_ solution with a pH of 11.0 after 0 min, 10 min, 20 min, 30 min, 40 min, 50 min, and 60 min, respectively.

Simulated intestinal fluid (SIF) was prepared according to He [21] with minor modifications. First, 10 g of trypsin and 6.8 g of KH_2_PO_4_ were dissolved in a moderate amount of water. The pH was adjusted to 6.8 with 0.2 mol/L NaOH solution or 0.2 mol/L hydrochloric acid solution, and then diluted to 1000 mL with water. Then, the test lectin solution (5 mg/mL) was mixed with the SIF solution (pH 6.8) in a water bath to start the tryptic digestion reaction. The trypsin reaction was immediately terminated by heating to boiling after a certain time.

#### 2.5.5. Antibacterial Experiment of Zihua Snap Bean Lectin

Antibacterial activity was investigated by the disc diffusion method [30]. *S. aureus*, *E. coli*, and *B. subtilis* were used. Bacteria were grown in shaker flasks containing potato medium and placed at 28 °C for 24 h. After diluted to 10^7^ colony-forming units/mL (CFU/mL), 100 µL of bacterial suspension was smeared onto the surface of an inoculated medium. Four filter papers with diameters of 5 mm were placed on the surface of the inoculated medium of the plate. Then, 20 μL of lectin solution with concentrations of 0.5 mg/mL, 1 mg/mL, and 2.5 mg/mL was added to the filter papers, respectively. A stroke-physiological saline solution was used as a blank control. Then, 20 μL of phenol solution with concentrations of 0.5 mg/mL, 1 mg/mL, and 2.5 mg/mL was used as a positive control. After being incubated at 28 °C for 12 h, the plate was taken out for observation and recording. A transparent ring around the filter paper revealed antimicrobial activity.

#### 2.5.6. Antifungal Experiment of Zihua Snap Bean Lectin

*P. infestans* was carried out in a petri plate containing tomato juice agar and incubated in a dark incubator at 22 °C for seven days. After the mycelial colony had developed, two filter papers with diameters of 5 mm were placed in front of the mycelial colony. Then, 20 μL of lectin solution (5 mg/mL) and 20 μL of stroke-physiological saline solution were added to the two filter papers, respectively. The plate was incubated at 22 °C for seven days and taken out of the plate for observation.

### 2.6. Electrophoresis

SDS-PAGE was used to identify lectin and evaluate the digestion of lectin by 12% bis-acrylamide homogeneous gel. The gel was run for 95 min using TRIS–glycine–SDS running buffer at constant voltages of 80 V for the stacking gel and 120 V for the separating gel. After the electrophoresis was run, the gel was washed with several volumes of distilled water for several times, and then stained for 30 min with Coomassie brilliant blue R-250 and destained with destaining solution. 

### 2.7. Nano LC-ESI-MS/MS Analysis

NanoLC-ESI-MS/MS analysis of the digested lectin sample was carried out by a high-pressure liquid chromatography (HPLC) system (Agilent, Palo Alto, CA, USA) with an Agilent C18 column (75 μm × 8 cm, 3 μm). Mobile phase A consisted of 97.5% water, 2% acetonitrile, and 0.5% formic acid, whereas mobile phase B consisted of 9.5% water, 90% acetonitrile, and 0.5% formic acid. The gradation time from 2% mobile phase B to 90% mobile phase B was 60 min, plus 20 min for sample loading and 20 min for column washing. The injection volume was 3 μL. The HPLC system was online coupled with a linear ion trap mass spectrometer (LTQ, Thermo, San Diego, CA, USA) in a way that a sample eluted from an HPLC column was directly ionized by an electrospray ionization (ESI) process and entered into the mass spectrometer. The ionization voltage was often optimized in the instrument-tuning process, and normally in a range of 1.5kv to 1.8kv. The capillary temperature was set at 100 °C. The mass spectrometer was set at the data-dependent mode to acquire MS/MS data via a low-energy collision-induced dissociation (CID) process. The default collision energy was 33%, and the default charge state was three. One full scan with one microscan with a mass range of 350 amu to 1650 amu was acquired, followed by nine MS/MS scans of the nine most intense ions with a full mass range and three microscans. The dynamic exclusion feature was set as following: repeat count of one and an exclusion duration of 1 min. The exclusion width was 4 Da. The mass spectrometric data was used to search against the most recent non-redundant protein database (NR database, NCBI (Wellington, DE, USA)) with ProtTech’s ProtQuest (Philadelphia, PA, USA) software suite.

### 2.8. Fourier Transform Infrared Sectroscopy

The infrared spectrum of the Zihua snap bean lectin was obtained with a Fourier transform infrared spectrometer (Shimadzu, Kyoto, Japan) within the wave number range of 400 to 4000 cm^−1^. First, 2 mg of the tested lectin lyophilized powder were ground together with 20.0 mg of spectroscopic grade KBr powder and pressed into a 1-mm pellet. The experiment was repeated three times. The spectra was processed using Peakfit v4.12 software (SeaSolve, San Jose, CA, USA). 

### 2.9. Fluorescence Spectroscopy 

The lectin lyophilized powder was dissolved in 10 mmol/L of phosphate buffer (pH 7.2) and measured with a fluorescence spectrometer (PerkinElmer LS55, Fremont, CA, USA) at fluorescence excitation wavelengths of 280 nm and 295 nm, respectively. The emission spectrum were recorded between 300–400 nm. The slit widths of the excitation and emission monochromators were set to 5 nm. Fluorescence spectral baseline subtraction was performed using a sample buffer system as a blank control.

### 2.10. Statistical Analysis

The data were expressed as the mean or the percent mean ± standard deviation (SD). All the experiments were tested and analyzed in triplicate. An analysis of variance (ANOVA) was identified to determine the significant differences (*P* < 0.05) between means. 

## 3. Results and Discussion

### 3.1. Purification of Zihua Snap Bean Lectin

**Figure 1:**
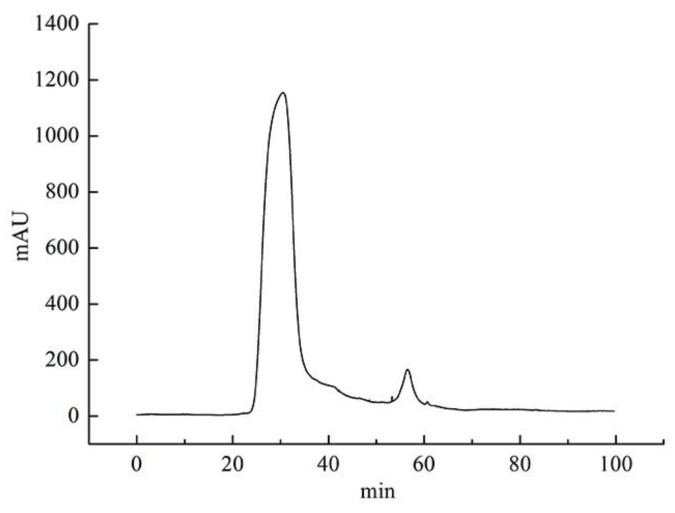
Gel chromatography of lectin extracted by an aqueous two-phase system (ATPS) on Sephadex G-75.

**Figure 2:**
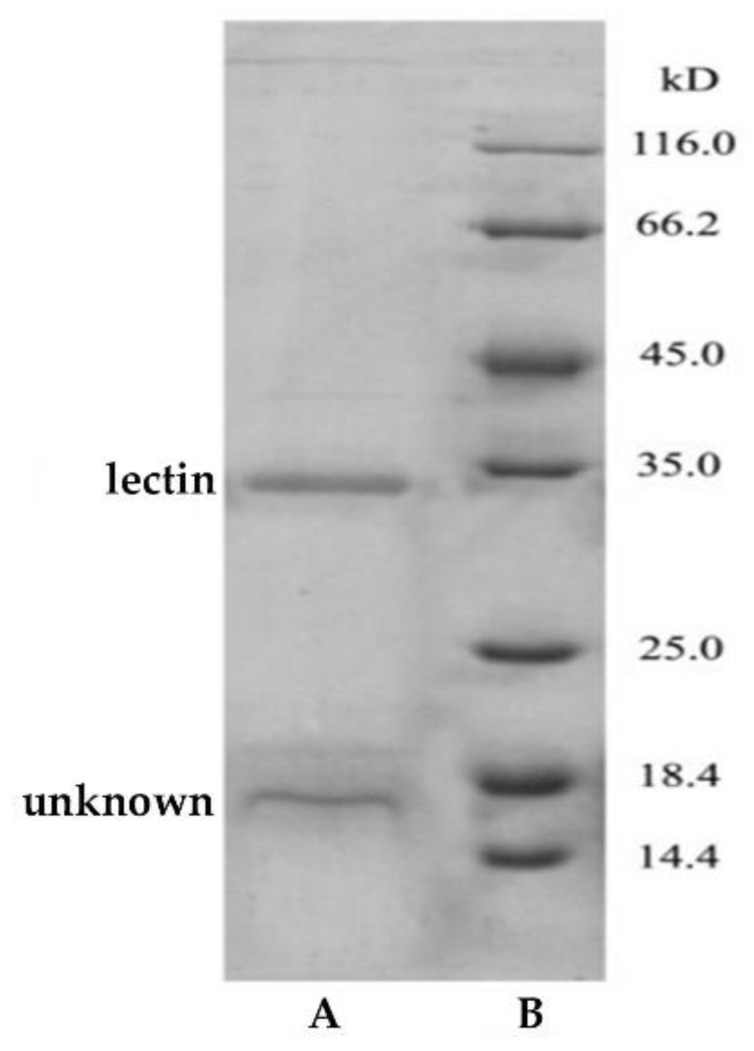
SDS-PAGE of the lectin after Sephadex G-75 purification: **A**, sample; **B**, marker.

The elution curve of the lectin purified by a two-step method based on an aqueous two-phase system and Sephadex G-75 column was shown in Figure 1. As indicated in Figure 1, two peaks could be obtained on the elution curve. Solutions corresponding to the two peaks were collected for the hemagglutinating test, and it was found that the solution that corresponded to peak 1 showed hemagglutinating activity, while the solution that corresponded to peak 2 did not. The active solution was collected and dialyzed to remove salts, and then subjected to SDS-PAGE. The electrophoresis result is shown in Figure 2.

As indicated in Figure 2, a band (lectin tape) at 35 kDa and a band (unknown tape) at 18.4 kDa were found in the SDS-PAGE of the lectin after Sephadex G-75. The SDS-PAGE suggested that the lectin existed as a monomer with a molecular weight of about 35 kDa, which is similar to that obtained by Jiang [25]. It was conjectured that the band of 18.4 kDa might be due to the partial dissociation of the lectin subunit. Compared to the band at 18.4 kDa, the band at 35 kDa may be due to the incomplete unfolding of the non-aggregated lectin that occurs in the presence of SDS denaturing conditions [31]. The band, unknown tape, at 18.4 kDa was not sure. We defined it as an unknown sample for mass spectrometric analysis.

### 3.2. Identification of Zihua Snap Bean Lectin by Tandem Mass Spectrometry

The lectin sample tape and the unknown sample tape in Section 3.1 were subjected to liquid chromatography-nano electrospray ionization mass spectrometry (Nano LC-ESI-MS/MS), respectively.

#### 3.2.1. Lectin Tape Analysis

The database UniProt was used to analyze the peptides hydrolyzed by sequencing grade modified trypsin (Promega). The molecular weight of the Zihua snap bean lectin was 29,742.25 Da, and its relative abundance was 57.3%. There were also other proteins, most of which were undetermined, as shown in Table 1. In addition, a few were α-amylase inhibitors with a molecular weight of 27,190.47 Da and a relative abundance of 6.1%.

**Figure 3:**
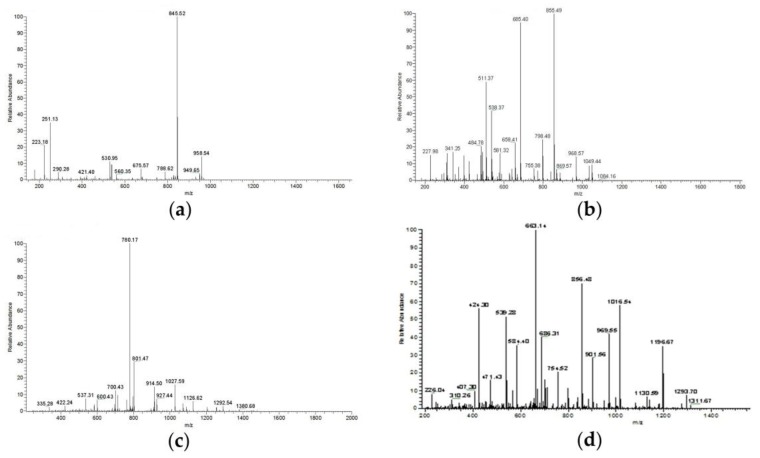
Two level mass spectrometry of the Zihua snap bean lectin (**a**) HIGIDVNSIK, (**b**) GGLLGLFNNYK, (**c**) GENAEVLITYDSSTK, and (**d**) DKGGLLGLFNNYK.

Figure 3 showed the peptides in the Zihua snap bean lectin. As indicated in Figure 3b, the peak *m*/*z* 855.49 was the strong peak of the y cleavage peptide GLFNNYK, while the peak *m*/*z* 685.40 was the stronger peak of the y cleavage peptide FNNYK. The peak *m*/*z* 511.32 was the weaker peak of the b cleavage peptide GGLLG. The peak *m*/*z* 227.98, the peak *m*/*z* 341.25, the peak *m*/*z* 658.41, and the peak *m*/*z* 1049.44 corresponded to the b cleavage peptides GGL, GGLL, and GGLLGLFNNY, respectively. The peak *m*/*z* 798.48 and the peak *m*/*z* 968.57 were y cleavage peptides LFNNYK and LGLFNNYK, respectively. As shown in Figure 3d, the peak *m*/*z* 856.48 [M+H] + was the stronger peak of the y cleavage peptide, GLFNNYK, and the peak *m*/*z* 686.31 [M+H] + was stronger peak of the y cleavage peptide, FNNYK. The identified peptides of the Zihua snap bean lectin are shown in Table 2, which indicated that the peptides of the two primary cleavages of lectin were GGLLGLFNNYK and DKGGLLGLFNNYK, respectively. The two peptides had overlapping portions to obtain a complete amino acid sequence, which proves that the protein was a lectin.

#### 3.2.2. Unknown Sample Tape Analysis

The results of Table 3 showed that 34.4% of relative abundance was still a lectin with a molecular weight of 29,742.25 Da, meaning that unknown sample band with 18 KDa was due to the partial dissociation of the lectin subunit.

It could be obtained from Table 2 and Table 4 that the identified peptides in the unknown samples had fewer species than the identified peptides in the lectin samples. Nano LC-ESI-MS/MS mass spectrometry showed that the molecular weight of the Zihua snap bean lectin was 29,742.25 Da, and the two-step method based on an aqueous two-phase system and Sephadex G-75 was suitable to separate and purify lectin.

### 3.3. The Properties of Zihua Snap Bean Lectin

#### 3.3.1. Carbohydrate Specificity of Zihua Snap Bean Lectin

Glucose, fructose, sucrose, d-maltose, d-trehalose, *N*-acetyl-d-glucosamine, *N*-acetyl-d-galactosamine, d-galactose, and lactose were used to study the carbohydrate specificity of the Zihua snap bean lectin. None of the nine glycosyl groups tested could inhibit the hemagglutinating activity of the Zihua snap bean lectin. Consistent with the result, d-fructose, d-glucose, and sucrose failed to inhibit the hemagglutinating activity of deep red kidney bean lectin [23]. As for *Hypsizigus marmoreus* lectin, glucose, mannose, L-fucose, and lactose had no inhibitory effect [32]. According to related literature, the glycosyl group specifically bound by some of the bean lectin is a polysaccharide with a larger molecular weight than a monosaccharide or oligosaccharide [33].

#### 3.3.2. Metal Ions’ Dependency of Zihua Snap Bean Lectin

It was confirmed that certain metal ions played a key role in maintaining the stability of the lectin structure and maintaining its specific biological activity [34]. Ethylenediaminetetraacetic acid (EDTA) is a divalent metal ion-chelating agent. When the lectin fully reacted with EDTA, the hemagglutinating activity of lectin was lost, and the reaction was generally reversible [35]. 

After fully reacting with EDTA, the hemagglutinating activity of the lectin was completely lost. The effect of metal ions on the recovery of the hemagglutinating activity was studied by adding different metal ions containing Cu^2+^, Na^+^, Mn^2+^, Ba^2+^, Mg^2+^, K^+^, Fe^2+^, Fe^3+^, and Ca^2+^. The results showed that Cu^2+^, Na^+^, Ba^2+^, K^+^, Fe^2+^, and Fe^3+^ ions had no effect on restoring the hemagglutinating activity of the lectin. Meanwhile, among the ions that could restore the Zihua snap bean lectin activity, Mn^2+^ and Ca^2+^ ions could completely restore the hemagglutinating activity of the lectin, while Mg^2+^ ions could only partially restore the activity. Some researchers indicated that the agglutinating activity of *Inocybe umbrinella* lectin was inhibited by Ca^2+^, Mg^2+^, and Mn^2+^ ions, but was unaffected by Fe^3+^ ions [36]. In addition, Ca^2+^ ions showed a significant effect on the agglutinating activity of lectin from *Laetiporus sulphureus* mushroom [37]. It could be concluded that Mn^2+^ and Ca^2+^ ions were essential metal ions of the Zihua snap bean lectin and played an important role in maintaining its biological activity, while Mg^2+^ ions could play a certain substitution role. Previous studies indicated that when Mn^2+^ and Ca^2+^ ions in proteins were chelated with EDTA, the protein conformation of lectin gradually changed to an open conformation. When a specific metal ion recombines with a lectin, the lectin slowly changed to a binding conformation. The change of binding conformation and open conformation was a cis-trans isomerization process, and the biological activity of lectin depended on the resulting conformations [26].

#### 3.3.3. Thermal Stability of the Zihua Snap Bean Lectin 

##### 3.3.3.1. Infrared Analysis

**Figure 4:**
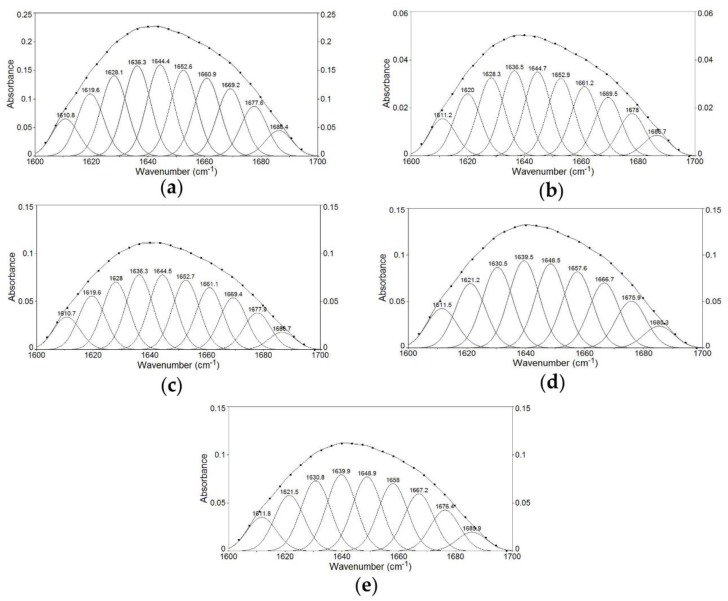
Second-derivative Fourier transform infrared (FTIR) spectra in the amide Ι region and Gaussian curve fitting of lectin at (**a**) room temperature, (**b**) 70 °C, (**c**) 80 °C, (**d**) 90 °C, and (**e**) 100 °C.

FTIR was used to study the changes of the secondary structure of the Zihua snap bean lectin during thermal treatment. The second derivative of the amide Ι region of the lectin treated at different temperatures (70 °C, 80 °C, 90 °C, and 100 °C) was found, and the Gauss peak shape was used for fitting. Different bands that overlapped together were analyzed, and the results are shown in Figure 4.

The corresponding relationship between each sub-peak and secondary structure meant that ~1610–1642 cm^−1^ was a β-fold structure; ~1642–1650 cm^−1^ was an irregular curly structure; ~1650–1660 cm^−1^ was an α-helix structure; ~1660–1680 cm^−1^ was the β-turn structure; and ~1680–1700 cm^−1^ was the β-reverse structure [38]. According to the corresponding relationship, the relative percentages of various secondary structures of lectin were obtained. As shown in Table 5, in the unheated lectin, the β-fold was the most abundant secondary structures, with the relative percentages of 40.43%. As the temperature increased, the α-helix content decreased, while the irregular curl content increased, and the most significant change was obtained at 90 °C (*p* < 0.05, n = 3). It indicated that the lectin started to depolymerize under thermal treatment, and the random coil structure was mainly converted from the β structure.

##### 3.3.3.2. Fluorescence Spectroscopy Analysis 

**Figure 5:**
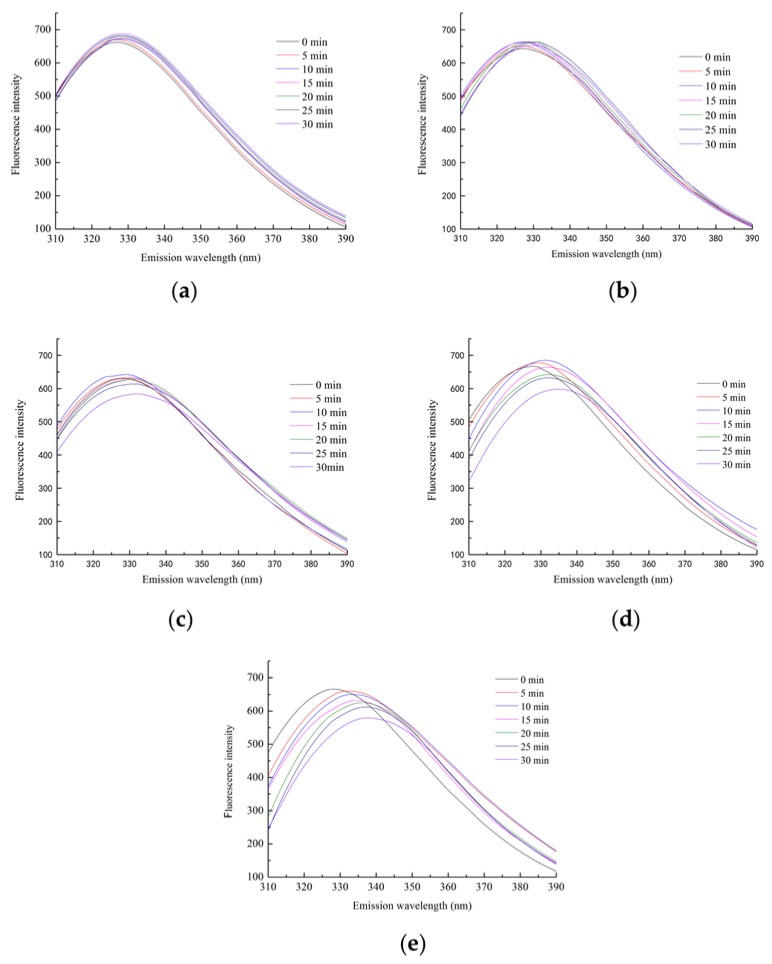
The intrinsic fluorescence spectra of lectin at (**a**) 50 °C, (**b**) 60 °C, (**c**) 70 °C, (**d**) 80 °C, and (**e**) 90 °C.

**Figure 6:**
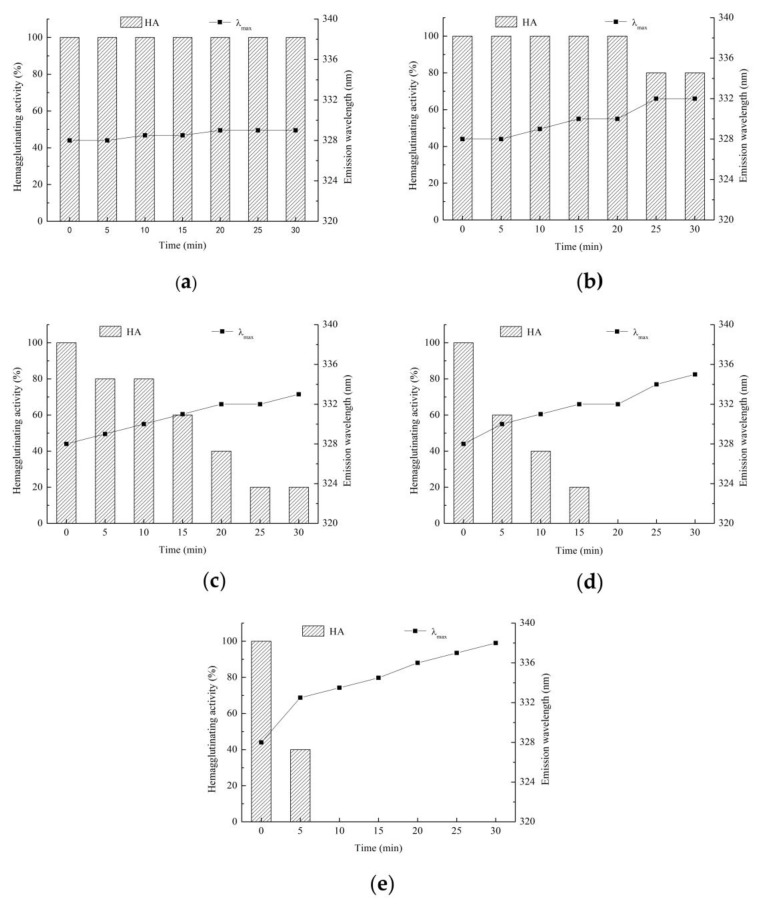
Hemagglutinating activity changes of lectin at (**a**) 50 °C, (**b**) 60 °C, (**c**) 70 °C, (**d**) 80 °C, and (**e**) 90 °C.

Fluorescence spectroscopy could reflect the changes in the tertiary structure of proteins [39]. The maximum emission wavelength of the native lectin (0 min) was maintained between 328–330 nm in Figure 5. All the tryptophan residues in lectin might be buried inside the hydrophobic cavity of the protein [29]. As shown in Figure 5a,b, when the heating time was short or the heating temperature was low, the fluorescence intensity was basically unchanged, and the hemagglutinating activity of lectin was not obviously affected (Figure 6a,b). As shown in Figure 5c–e, fluorescence intensity increased when the heating time was long or the heating temperature was high, which might be related to the thermal polymerization of lectin multimers caused by heat treatment [40]. In addition, there was a red shift in maximum emission wavelength (Figure 6c–e). It represented a change in the microenvironment of the tryptophan residue, which indicated that the tryptophan residue was transferred to a hydrophilic environment [41]. As the heating temperature reached 90 °C, the fluorescence intensity decreased most significantly, and the hemagglutinating activity of lectin dropped sharply, with all activity lost within 10 min (Figure 6e). In addition, the maximum emission wavelength of the tryptophan residue was accompanied by a further red shift to 340 nm (Figure 5e). The maximum emission wavelength of tryptophan residues in the hydrophilic environment was 350–360 nm. It indicated that the lectin folding structure was not fully expanded, and the lectin had good heat resistance. 

#### 3.3.4. pH Stability of Zihua Snap Bean Lectin 

**Figure 7:**
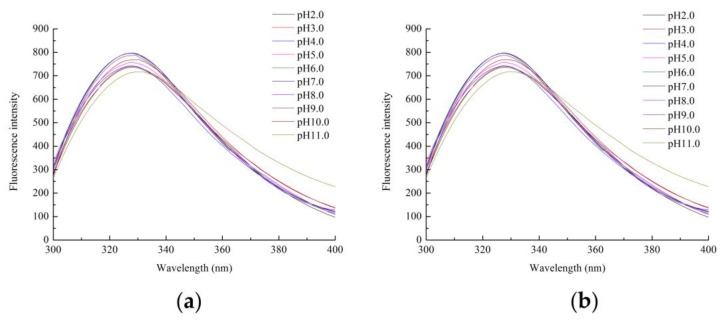
The emission fluorescence spectra of lectin from the Zihua snap bean at various pH conditions at the excitation wavelengths of (**a**) 280 nm and (**b**) 295 nm.

The endogenous fluorescence of the Zihua snap bean lectin at different pH conditions was measured at excitation wavelengths of 280 nm and 295 nm, respectively. The experimental results were shown in Figure 7. 

As shown in Figure 7a,b, as the pH increased in the range of 3.0 to 10.0, the fluorescence intensity gradually decreased, and the maximum absorption wavelength gradually increased. However, the change was not significant. The maximum emission wavelength of the Zihua snap bean lectin in the range of pH 3.0 to 10.0 was 329 ± 1.5 nm; there were a blue shift at 2.0 and a red shift at pH 11.0. It was indicated that most of the tryptophan residues in the lectin were in a non-polar environment. When the protein was fully expanded, the maximum emission wavelength of the exposed tryptophan residue was between 350–360 nm. Thus, the acid-induced development of the Zihua snap bean lectin did not cause the tryptophan residue to directly contact with water. The fluorescence intensity decreased at pH 2.0 and 11.0 in Figure 7. However, it cannot be explained simply by the denaturation of proteins in acidic or alkaline environments. The reduction of fluorescence intensity at low pH values may be caused by fluorescence quenching or the neutralization of COO– groups on acidic amino acids near the fluorophore [40]. In general, the fluorescence spectra of nectarines had a peak shift at lower or higher pH conditions, and the difference in the position of the peak shift was not significant. It was indicated that the lectin structure did not show significant differences within the tested pH range.

**Figure 8:**
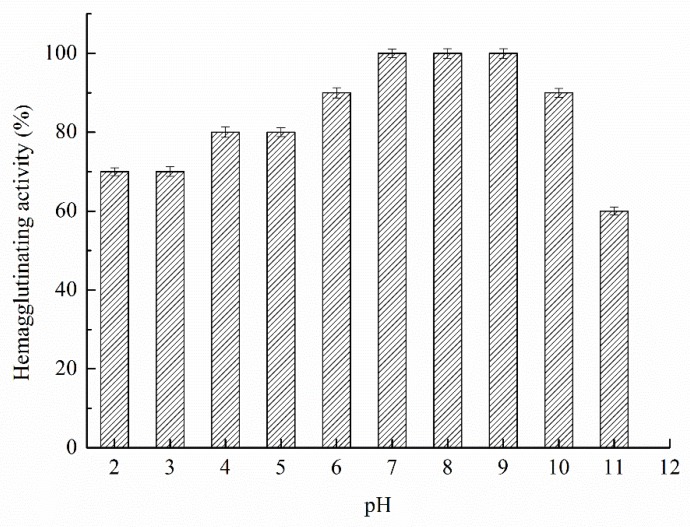
The hemagglutinating activity of the lectin from the Zihua snap bean at various pH conditions.

Figure 8 indicates that the Zihua snap bean lectin has hemagglutinating activity in a wide pH range (2.0 to 10.0). Unlike temperature and denaturant, pH-induced protein unfolding was achieved by protonation or relatively few discrete sites of protonation [42], whereas perturbing a small number of residues did not allow the protein to fully unfold. It has been reported that lectins maintain their tertiary structure stability mainly through non-covalent forces such as hydrogen bonding, ionic interaction, hydrophobic interaction, van der Waals force, and disulfide bond covalent linkage [43]. The change in the tertiary structure of the acid-induced Zihua snap bean lectin might be due to the interaction of ions or hydrogen bonds. However, this change had no effect on the hemagglutinating activity of the lectin. 

**Figure 9:**
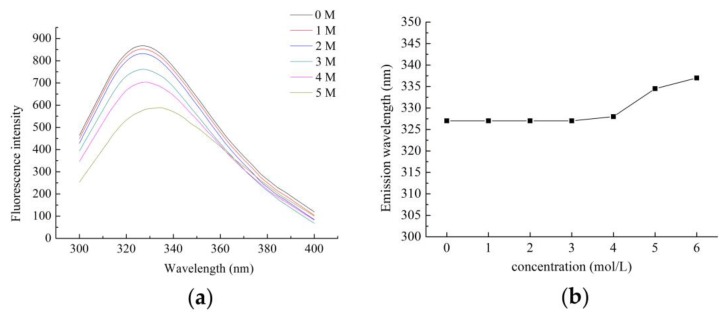
The fluorescence spectra (**a**) and maximum emission wavelength (**b**) of the lectin at different GdnHCl concentrations.

The pH stability of the Zihua snap bean lectin was studied using the denaturing agent guanidine hydrochloride (GdnHCl). Some models suggested that GdnHCl could migrate to the interior of the protein to form hydrogen bonds to reduce the hydrophobic effect of the protein [44]. Under physiological conditions of pH 7.2, the lectin reacted with different concentrations of GdnHCl for 24 h. The results of the conformational changes are shown in Figure 9. The fluorescence intensity gradually decreased with the progressive increase in GdnHCl concentration (Figure 9a). The maximum emission wavelength of lectin did not change obviously between 0–3 mol/L of GdnHCl, while the maximum emission wavelength of lectin showed a significant increase between 3–6 mol/L GdnHCl (Figure 9b). When the concentration of GdnHCl reached 6 mol/L, the fluorescence intensity decreased by two-thirds compared to 0 mol/L, and the maximum emission of lectin was a red shift to 337 nm, which was much smaller than the maximum emission wavelength (350–360 nm) of the tryptophan residue. It indicated that the tryptophan residue was not completely exposed to the solvent, and the folding structure of the lectin was not fully opened. The result showed that the acid–base stability of the Zihua snap bean lectin was high.

### 3.4. In Vitro Studies of the Digestibility of Zihua Snap Bean Lectin 

#### 3.4.1. The Digestibility of Native Lectin In Vitro

**Figure 10:**
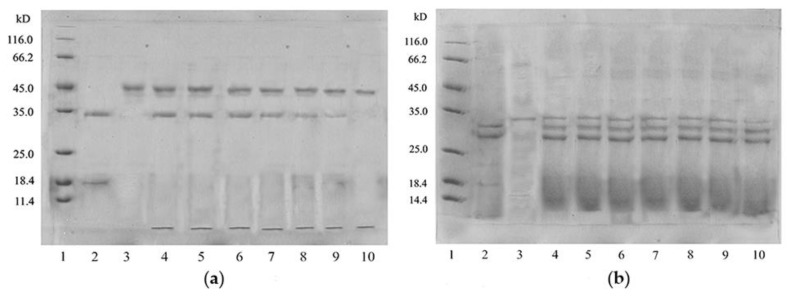
(**a**) Simulated gastric fluid (SGF) digestion profiles of the native lectin from the Zihua snap bean. In the SDS-PAGE analysis, lane 1 was the molecular weight marker, lane 2 was the native lectin, lane 3 was the pepsine, and lanes 4 to 10 were the SGF digestion pattern of the native lectin at 0 min, 2 min, 5 min, 10 min, 20 min, 30 min, and 60 min. (**b**) Simulated intestinal fluid (SIF) digestion profiles of the native lectin from the Zihua snap bean. In the SDS-PAGE analysis, lane 1 was the molecular weight marker, lane 2 was the native lectin, lane 3 was the tryptic, and lanes 4 to 10 were the tryptic digestion pattern of the native lectin at 0 min, 10 min, 20 min, 30 min, 40 min, 60 min, and 90 min.

Since the Zihua snap bean lectin can agglutinate rabbit red blood cells, it is probably a sensitizing protein. Therefore, the in vitro digestion simulation experiment of the Zihua snap bean lectin was studied. SDS–PAGE has been widely used in simulated gastric fluid (SGF) analysis and simulated intestinal fluid (SIF) analysis [45]. SDS–PAGE analyses of native lectin from the Zihua snap bean in SGF and SIF are shown in Figure 10.

As indicated in Figure 10a, as the digestion time prolonged, the native lectin from the Zihua snap bean was gradually decreased in SGF. It was difficult to observe the lectin band at 60 min. The results of the in vitro simulated digestibility evaluation of food proteins indicated that most food allergens were basically stable during SGF for 60 min [46]. Therefore, the Zihua snap bean lectin had a certain anti-enzymatic ability in the gastrointestinal tract, which laid a foundation for the application of lectin in the field of medicine. 

As shown in Figure 10b, under the tryptic digestion conditions, there was still a clear lectin fragment that remained until 90 min. A lectin from the red kidney bean band could still be observed after 48 h in SIF [47]. It indicated that the native lectin had good stability. The stability of the lectin provides an estimate of whether the protein may trigger clinical symptoms of an allergic disease.

#### 3.4.2. The Digestibility of Thermal-Treated Lectin

**Figure 11:**
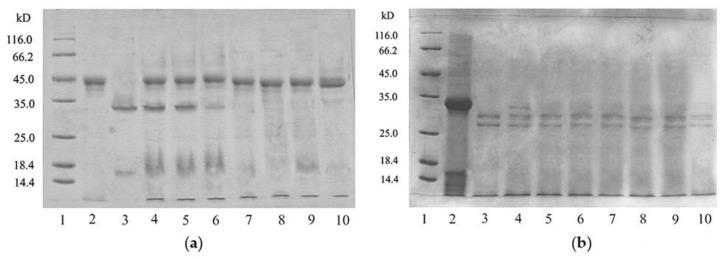
(**a**) SGF digestion profiles of the preheated lectin from the Zihua snap bean. In the SDS-PAGE analysis, lane 1 was the molecular weight marker, lane 2 was the pepsine, lane 3 was the native lectin, and lanes 4 to 10 were the SGF digestion pattern of the native lectin at 0 min, 2 min, 5 min, 10 min, 20 min, 30 min, and 60 min. (**b**) SIF digestion profiles of the preheated lectin from the Zihua snap bean. In the SDS-PAGE analysis, lane 1 was the molecular weight marker, lane 2 was the tryptic, lane 3 was the native lectin, and lanes 4 to 10 were the tryptic digestion pattern of the native lectin at 0 min, 10 min, 20 min, 30 min, 40 min, 60 min, and 90 min.

In vitro digestion experiments were performed on the preheated lectin from the Zihua snap bean. The results are shown in Figure 11.

The preheated zihua snap bean lectin could be completely digested by pepsin in SGF in 10 min (Figure 11a). Similarly, no obvious lectin bands were observed in SIF after 10 min (Figure 11b). It meant that the digestibility of lectin from the Zihua snap bean was changed to some extent by thermal treatment. Some studies proved that the partial structure of lectin after preheating treatment was unfolded, which was helpful to improve the in vitro enzymatic hydrolysis of lectins [16].

### 3.5. Antimicrobial of Zihua Snap Bean Lectin

#### 3.5.1. Antibacterial Activity of Zihua Snap Bean Lectin

**Figure 12:**
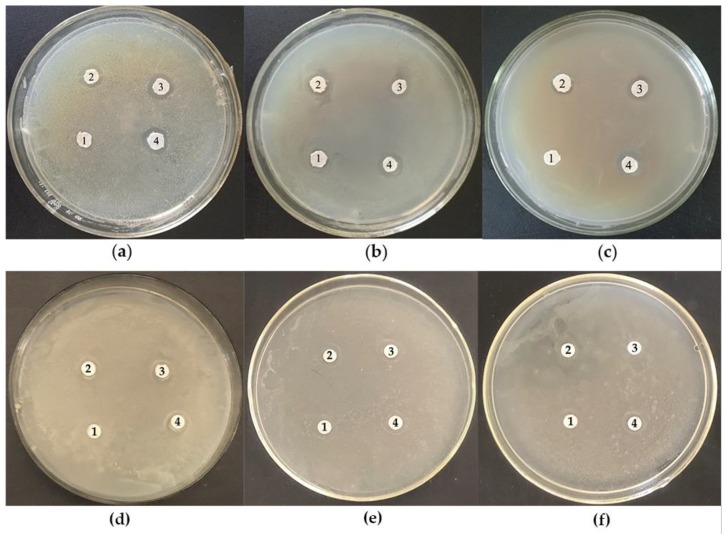
Inhibition of (**a**) *S. aureus*, (**b**) *E. coli*, and (**c**) *B. subtilis* with different concentrations (1, stroke-physiological saline solution; 2, 10 µg; 3, 20 µg; 4, 50 µg) of lectin. Inhibition of (**d**) *S. aureus*, (**e**) *E. coli*, and (**f**) *B. subtilis* with different concentrations (1, stroke-physiological saline solution; 2, 10 µg; 3, 20 µg; 4, 50 µg) of phenol.

The inhibition of *S. aureus*, *E. coli,* and *B. subtilis* by the Zihua snap bean lectin is shown in Figure 12a–c, respectively. Figure 12d–f were the positive control of phenol. The in vitro antibacterial experiment indicated that the Zihua snap bean lectin showed antibacterial activity against the tested bacteria (*S. aureus*, *E. coli*, and *B. subtilis*). Lectins have the ability to recognize carbohydrates (e.g., peptidoglycan and lipopolysaccharide) on the surface of bacterial cells [48]. The antibacterial activity of lectins was attributed to their interaction with the glycans of the bacterial cell wall [49]. In addition, antibacterial lectins can promote protein leakage and the formation of pores in the cell wall [50]. As shown in Figure 12, *S. aureus*, *E. coli,* and *B. subtilis* were capable of being inhibited by the lectin. At the same concentration, the antibacterial effect of lectin was stronger than that of phenol. The corresponding diameter of inhibition halos was shown in Table 6. As the lectin addition amount increased, the inhibition halo grew larger. Romero et al. [30] indicated that *Phthirusa pyrifolia* leaf lectin (PpyLL) had an inhibitory effect on *B. subtilis*, but not on *S. aureus*, when the addition amount was 80 µg. Lectin from the seeds of *Archidendron jiringa* Nielsen was detected to have inhibition for *B. subtilis* and *S. aureus*, whereas it did not for *E. coli* [51]. Some studies have shown that the lectin had a greater inhibition on Gram-positive bacteria than Gram-negative bacteria [30,51]. Our results agreed with this observation. The difference of the peptidoglycan content of cell walls of Gram-positive bacteria and Gram-negative bacteria was the main cause of this condition. 

#### 3.5.2. Antifungal Activity of Zihua Snap Bean Lectin

**Figure 13:**
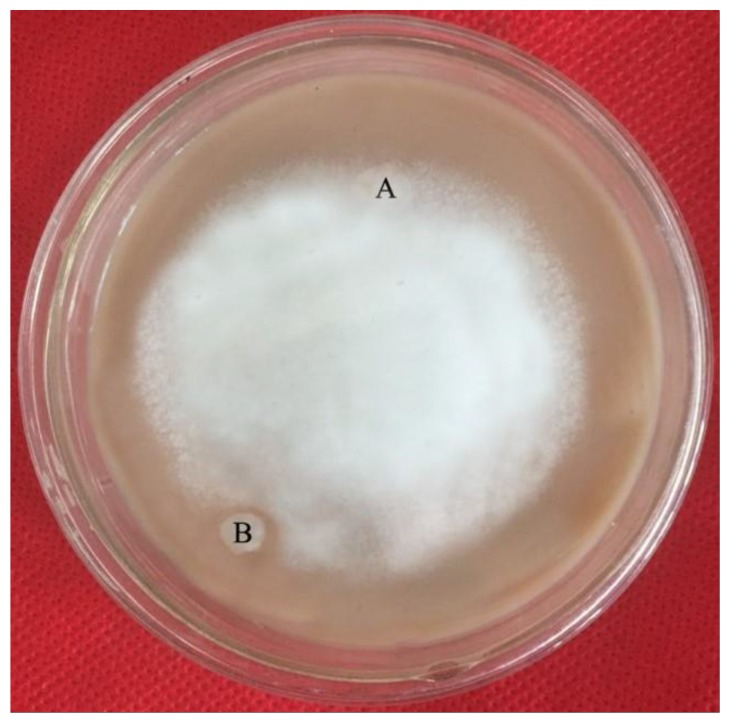
Inhibition of the *P. infestans* with lectin (**B**) and stroke-physiological saline solution (**A**).

As indicated in Figure 13, the Zihua snap bean lectin showed an inhibitory effect on *P. infestans* when the addition amount was 100 μg (20 μL of 5 mg/mL lectin solution). Compared with the results of antibacterial experiments, the Zihua snap bean lectin can inhibit the growth of *P. infestans* at a high concentration. According to de Santana [35], only a few lectins had significant antifungal activity. In addition, lectins from kidney beans have inhibitory effects on four harmful fungi (*Helminthosporium maydis*, *Sclerotinia sclerotiorum*, *Gibberalla sanbinetti,* and *Rhizoctonia solani*) in agriculture [52]. Lectins interacting with chitin, α-mannan, and β-glucan in the cell walls can lead to growth inhibition, cell wall disruption, reduced absorption of nutrients, and interference with spore germination, which is the main reason for the antifungal activity of lectins [53].

## 4. Conclusions

The properties of the lectin isolated and purified by the two-step method were studied in this study. Through the experimental study on the affinity characteristics of lectin, it found that glucose, *N*-acetyl-d-glucosamine, d-galactose, *N*-acetyl-d-galactosamine, fructose, sucrose, d-maltose, d-trehalose, and lactose could not inhibit the hemagglutinating activity of the lectin and Mn^2+^, Ca^2+^, and Mg^2+^ ions can restore the hemagglutinating activity of the lectin. The results of the thermal stability indicate that the hemagglutinating activity of the lectin is changed with the change of protein conformation. Studies on the pH stability of the lectin show that the lectin maintains its hemagglutinating activity in the range of pH 2.0 to 10.0. The SDS-PAGE of the in vitro digestion found that the native lectin is almost completely digested by pepsin at 60 min in SGF, while a distinct lectin band is observed in SIF for 90 min. However, the lectin pretreated is completely digested by pepsin or trypsin in about 10 min. Antibacterial assays demonstrate that the lectin exhibits antibacterial activity against *S. aureus*, *E. coli,* and *B. subtilis*. In addition, the lectin shows inhibition on the growth of phytophthora infestans at a high concentration.

## Figures and Tables

**Table 1:** The test results of the lectin sample by liquid chromatography-nano electrospray ionization mass spectrometry (Nano LC-ESI-MS/MS).

Hits	Protein Molecular Weight/Da	Number of Peptides	Link	Relative Abundance
1	29,742.25	246	Q8RVX6	57.3%
2	39,105.93	65	V7BFT4	8.6%
3	35,564.65	56	V7AIB2	10.8%
4	27,190.47	27	P02873	6.1%
5	36,314.02	26	V7BPP1	2.9%
6	97,527	26	V7BX14	1.6%
7	97,769.06	23	V7BZK0	0.6%
8	71,495.53	20	V7C9P5	0.1%

**Table 2:** The peptide of the Zihua snap bean lectin as determined by Nano LC-ESI-MS/MS.

Peptide Number	Amino Acid Sequence	*m*/*z*
6046	HIGIDVNSIK	1094.61
6339	TTTWDFVK	996.49
6350	LTNVNDNGEPTLSSLGR	1785.89
7217	DKGGLLGLFNNYK	1437.76
7269	GNVETNDVLSWSFASK	1752.83
7304	YDSNAHTVAVEFDTLYNVHWDPKPR	2973.40
7329	GGLLGLFNNYK	1194.64
7503	TTTWDFVKGENAEVLITYDSSTK	2604.26
8487	SVLPEWVIVGFTATTGITK	2018.11
8669	YDSNAHTVAVEFDTLYNVHWDPK	2720.25
8877	LSDGTTSEALNLANFALNQIL	2204.13
9249	LLVASLVYPSLK	1301.80
11054	DKGGLLGLFNNYK	1437.76

**Table 3:** The test results of unknown sample by Nano LC-ESI-MS/MS.

Hits	Protein Molecular Weight/Da	Number of Peptides	Login ID	Relative Abundance
1	29,742.25	104	gi|19773404|	34.4%
2	24,778.15	48	gi|2780981|	18.4%
3	50,723.5	29	gi|75708857|	3.1%
4	49,241.02	25	gi|130169|	2.8%
5	49,483.71	19	gi|351727923|	0.3%
6	71,980.77	17	gi|356500683|	0.4%
7	97,136.89	16	gi|351727843|	1.1%
8	47,824.05	15	gi|374297913|	0.7%

**Table 4:** The peptide of the lectin in an unknown sample as determined by Nano LC-ESI-MS/MS.

Peptide Number	*m*/*z*	Amino Acid Sequence
6346	1094.61	HIGIDVNSIK
6451	1785.89	LTNVNDNGEPTLSSLGR
6580	996.49	TTTWDFVK
6741	1246.67	FNETNLILQR
6991	1323.69	TSFIVSDTVDLK
7279 7326	1752.83 1437.76	GNVETNDVLSWSFASK DKGGLLGLFNNYK
7410	1301.80	LLVASLVYPSLK
7444	2973.40	YDSNAHTVAVEFDTLYNVHWDPKPR
7496	1194.64	GGLLGLFNNYK
8307	2018.11	SVLPEWVIVGFTATTGITK
8634	2204.13	LSDGTTSEALNLANFALNQIL

**Table 5:** The content of the secondary structure of lectin at different temperature.

Temperature	α-Helix	β-Fold	β-Turn	β-Reverse	Random Curl
25 °C	12.85 ± 0.14 ^b^	40.43 ± 0.11 ^a^	29.22 ± 0.12 ^d^	3.85±0.08 ^c^	13.60 ± 0.16 ^a^
70 °C	12.84 ± 0.13 ^b^	42.23 ± 0.08 ^d^	27.68 ± 0.17 ^c^	3.39±0.11 ^b^	13.64 ± 0.15 ^a^
80 °C	12.6 ± 0.19 ^b^	42.68 ± 0.13 ^e^	27.87 ± 0.15 ^c^	3.23±0.13 ^ab^	13.69 ± 0.08 ^a^
90 °C	11.65 ± 0.17 ^a^	41.74 ± 0.15 ^c^	25.59 ± 0.14 ^b^	3.13±0.14^a^	17.87 ± 0.11 ^b^
100 °C	11.58 ± 0.11 ^a^	40.76 ± 0.19 ^b^	23.86 ± 0.09 ^a^	3.82±0.17 ^c^	19.98 ± 0.19 ^c^

Note: The same letter followed by the same column means that the difference is not significant (*p* > 0.05), and the difference between marked letters indicates that the difference is significant (*p* < 0.05).

**Table 6:** The bacteriostatic circle diameter of bacteria with different concentrations of lectin.

Bacterial	Different Addition Amount (µg)	Inhibition Halo (mm)
*S. aureus* (G^+^)	10	10.5 ± 0.5
	20	12.1 ± 0.8
	50	14.5 ± 1.1
*E. coli* (G^−^)	10	8.2 ± 0.8
	20	10.1 ± 0.8
	50	11.6 ± 0.5
*B. subtilis* (G^+^)	10	8.8 ± 1.0
	20	10.70 ± 0.5
	50	12.7 ± 0.7
